# Barriers and Facilitators to Increased Parental, Caregiver, and Community Engagement in Obesity Prevention Targeting Vulnerable Children: A Qualitative Study in Greece

**DOI:** 10.3390/healthcare14050620

**Published:** 2026-02-28

**Authors:** Theodora Balafouti, Vaios Svolos, Matzourana Argyropoulou, Renos Roussos, Dimitra Eleftheria Strongylou, Christina Mavrogianni, Anela Halilagic, Sofia Koukouli, George Moschonis, Yannis Manios, Odysseas Androutsos, Theodora Mouratidou

**Affiliations:** 1 Department of Nutrition and Dietetic Sciences, School of Health Sciences, Hellenic Mediterranean University, 72300 Sitia, Greece; tbalafouti@hmu.gr (T.B.); ddk327@edu.hmu.gr (R.R.); 2 Institute of Agri-Food and Life Sciences, University Research & Innovation Center, Hellenic Mediterranean University, 71003 Heraklion, Greece; koukouli@hmu.gr (S.K.); manios@hua.gr (Y.M.); 3 Laboratory of Clinical Nutrition and Dietetics, Department of Nutrition and Dietetics, School of Physical Education, Sports Science and Dietetics, University of Thessaly, 42100 Trikala, Greece; vaiossvolos@gmail.com (V.S.); ntemi_st@hotmail.com (D.E.S.); oandroutsos@uth.gr (O.A.); 4 School of Medicine, Dentistry and Nursing, College of Medical, Veterinary and Life Sciences, University of Glasgow, Glasgow G12 8QF, UK; 5 European Centre for Obesity, Harokopio University, 17676 Athens, Greece; margyropoulou@hua.gr (M.A.); cmavrog@hua.gr (C.M.); g.moschonis@latrobe.edu.au (G.M.); 6 Department of Nutrition and Dietetics, School of Health Sciences and Education, Harokopio University, Kallithea, 17671 Athens, Greece; 7 Department of Food, Nutrition and Dietetics, School Allied Health, Human Services & Sport, La Trobe University, Melbourne, VIC 3086, Australia; a.halilagic@latrobe.edu.au; 8 Department of Social Work, School of Health Sciences, Hellenic Mediterranean University, 71410 Heraklion, Greece; 9 Laboratory of Interdisciplinary Approaches for the Enhancement of Quality of Life (Quality of Life Laboratory), Hellenic Mediterranean University, 71004 Heraklion, Greece

**Keywords:** health promotion, vulnerable children, healthy eating and physical activity

## Abstract

**Background/Objectives:** Social vulnerability is linked to unhealthy eating habits, low physical activity, and, overall, increased health risks and low well-being. This study examined self-perceived barriers and facilitators to engaging in obesity prevention policies for children at risk of poverty and social exclusion in Greece from the perspective of parents, caregivers, and community representatives. **Methods:** A qualitative study was conducted from November to December 2023 in three geographically diverse Greek regions, namely Attica, Thessaly, and Crete. A qualitative study was conducted between November and December 2023 in three geographically diverse regions of Greece. In total, seventy-two individuals participated in the study through individual interviews and focus groups. Forty-five parents of children with disabilities took part in individual interviews, equally represented in all three regions (fifteen participants per region). Among focus group participants: Twenty-one caregivers from child protection units participated in six focus groups (two per region), with focus group sizes ranging from three to five participants. In addition, six Roma community representatives participated in three focus groups (one per region), with focus group sizes ranging from one to four participants. Inductive and deductive thematic analysis were performed using NVivo 14 software to identify key themes. **Results:** Most factors that increased engagement were perceived by participants as both barriers and facilitators. These factors were classified at the individual, sociocultural, or structural level, and similar themes emerged across groups. Common barriers to poor engagement included low health literacy, financial difficulties and underfunding, social exclusion, a lack of targeted nutrition interventions, concerns related to training opportunities and support, and the adequacy and safety of built environments. Common facilitators of enhanced engagement included increased awareness and motivation to support vulnerable children, the availability of community- and school-based initiatives, and free school meal provision. **Conclusions:** Engagement in obesity prevention policies targeting vulnerable children is influenced by multiple interrelated factors. Understanding these barriers and facilitators from the participants’ perspectives can guide policymakers and practitioners in designing more effective obesity-related interventions for socially vulnerable groups of children.

## 1. Introduction

Social vulnerability refers to the conditions that put individuals or population groups at greater risk for negative health and well-being impacts due to socioeconomic, demographic, cultural, and other factors [1,2]. Children growing up in socially vulnerable environments face further disadvantages when their aforementioned life conditions are combined with factors such as poverty, low parental education, low household income, and parental unemployment. These children include those with disabilities, those from ethnic minorities (including Roma), those from migrant backgrounds, and those living in institutionalized care (defined as “children in need” according to the Recommendation for the European Child Guarantee (ECG) [3]. These conditions often restrict access to essential goods and services. They are also linked to obesogenic behaviors, such as unhealthy eating and physical activity patterns, and weight status [4,5,6], and limited access to health and social care [7,8]. According to recent research, health issues disproportionately affect children from vulnerable groups, including those from ethnic minorities, with disabilities, with mental health issues, living in child protection centers [9,10,11,12,13], and from low socioeconomic (SES) families [4,7,8,9,14,15]. According to the literature, in Greece, children from low SES families and rural areas are at a higher risk of overweight and obesity [4,9,14,15,16]. Previous studies have shown that vulnerable children whose parents are less educated, unemployed, or experiencing financial difficulties tend to be less aware of healthy behaviors, which can lead to unhealthy dietary patterns. Specifically, these children tend to consume fewer fruits and vegetables and more sugary drinks and processed foods, thereby increasing their risk of being overweight or obese [4,9,15]. Additionally, Greek children with disabilities are at an increased risk for obesity due to barriers to physical activity and maintaining healthy dietary habits [10]. Furthermore, children living in child protection units face significant nutritional and health risks, yet such data are lacking in Greece [13,17]. However, to date, evidence linking childhood obesity with categories of socially vulnerable children remains scarce, especially among children at risk of poverty and social exclusion, underscoring the need for tailored interventions to ensure inclusion and accessibility.

Childhood overweight and obesity rates in Greece are particularly alarming, with 37.5% of children aged 5 to 14 living with overweight or obesity, which is the highest rate in the EU [18]. Meanwhile, over one-third (33.6%) of children under 16 were at risk of poverty and social exclusion in 2024 [19]. Together, these underscore the burden of obesity and social vulnerability in childhood, emphasizing the urgent need for targeted national health policies and interventions to address these issues. In line with this, a recent scoping review of the national framework for childhood obesity found that of 28 policies, only one specifically addressed childhood obesity in the most vulnerable [20]. Furthermore, international frameworks reinforce this need. The WHO/EU “Ending Childhood Obesity by 2025” recommendations emphasize the importance of supportive nutrition, healthy environments, physical activity, and equitable access to healthcare for all children [21]. Similarly, the “UNICEF’s Nutrition Strategy 2020–2030” calls for a multisectoral, rights-based approach, combining prevention, early intervention, and regulatory measures to ensure that all children have access to nutritious food and supportive services [22]. To address such concerns, Greece implemented a recent ECG strategy targeting vulnerable children [3].

Effective development and implementation of childhood obesity interventions requires an in-depth understanding of target communities and their specific needs. While quantitative studies capture some general aspects, they do not necessarily fully reflect the community’s lived experiences or daily realities in addressing obesogenic environments and their respective determinants. Qualitative approaches are therefore essential in providing in-depth insights into the knowledge, experiences, and perceptions of factors influencing engagement in health promotion policies addressing childhood obesity [23], especially in communities where evidence is scarce. Therefore, it is important to capture the views of both policy implementers and beneficiaries, including parents and caregivers of children, as well as community representatives. Parents and caregivers play a vital role in managing childhood obesity, as their perceptions of their child’s weight strongly shape attitudes and behaviors towards prevention and management [11,24].

Findings from several previous qualitative studies exploring the perceptions and experiences of parents and stakeholders regarding childhood obesity revealed common challenges, including low parental health literacy, financial difficulties, challenges in promoting healthy behaviors, inadequate infrastructure and trained personnel, limited access to healthy foods in schools, and few incentives for physical activity. In contrast, facilitators included strong family and community support, health and nutrition literacy among implementers and beneficiaries, structured school or community initiatives, and supportive environments for healthy eating and physical activity [24,25,26]. Studies on children with disabilities highlighted additional barriers, such as social exclusion and negative societal attitudes, and the need to travel long distances to access appropriate physical activity. Facilitators included family and community support, peer involvement, targeted educational interventions, and suitable, safe infrastructure for physical activity [10,27].

Despite these insights, most research addresses the promotion of healthy eating and physical activity patterns, as well as childhood obesity, at a general level or within a single population group. Such research often uses socioeconomic status or educational attainment as indicators of social vulnerability. Consequently, there is a significant knowledge gap regarding the barriers and facilitators of obesity prevention interventions for children at risk of poverty and social exclusion, and the factors affecting engagement. This study aims to address this by exploring the self-perceived barriers and facilitators that influence engagement in obesity prevention interventions from the perspectives of parents, caregivers, and community representatives of socially vulnerable children. This will provide a more comprehensive understanding of the challenges and support relevant to this highly vulnerable population.

## 2. Materials and Methods

### 2.1. Study Design

A cross-sectional, multi-centered mixed-methods study was conducted as previously described [26]. A qualitative semi-structured approach was selected to explore lived experiences in depth and to identify perceived barriers and facilitators influencing engagement in obesity prevention policies, among beneficiaries, including parents, caregivers, and community representatives of socially vulnerable children, including those of Roma ethnic origin, those residing in child protection units, and those with disabilities. This approach enabled participants to freely share insights, facilitating an authentic understanding in cases where the topic was original and there was scarce scientific evidence [28].

Children at risk of poverty and social exclusion are defined in the European Union (EU) Council Recommendation 2021/1004 as “children in need”, including (a) homeless children or those experiencing severe housing deprivation; (b) children with disabilities; (c) children with mental health issues; (d) children with a migrant background or of minority ethnic origin, particularly Roma; (e) children in alternative care settings, primarily institutional care; and (f) children living in precarious family situations [29].

Focus groups were conducted with Roma community representatives and caregivers working in child protection units. Group interaction fosters valuable and rich data through participant discussions, often leading to new ideas and questions, while the group context promotes participant empowerment. Semi-structured individual interviews were conducted with parents of children with disabilities to ensure they felt comfortable addressing sensitive issues and to capture diverse perspectives relating to different types of disability and the associated challenges [28,30]. Data collection took place between November and December 2023 in three Greek regions: Attica, Thessaly, and Crete. These regions encompassed urban and rural settings, as well as diverse socioeconomic contexts.

### 2.2. Inclusion and Exclusion Criteria

Participants were required to live or work in Greece, speak and understand the Greek language, and provide informed consent. For the Rom focus groups, adult representatives of the community, capable of providing a broad community perspective (i.e., Roma community representatives), were recruited. Practical challenges such as language barriers and limited engagement in health-related issues within the community were considered [12]. Focus groups were therefore conducted with Roma community representatives, most of whom were also parents. While this approach may limit the transferability to the broader Roma population, this was mitigated by recruiting leaders from multiple regions. Caregivers in child protection units were eligible if they had been employed by the institution for at least one month and were directly or indirectly involved in childcare. Parents or caregivers of children with disabilities were eligible if they had at least one child with a disability, as defined by the WHO, across three dimensions: impairment in body structure or function or mental functioning, activity limitation, and restrictions in participation in normal daily activities. Those who did not meet the inclusion criteria, were unable to provide informed consent, or lacked direct experience related to the study objectives were excluded.

### 2.3. Procedure and Ethical Approval

A convenience sampling approach was employed to recruit participants who were readily accessible. Recruitment strategies varied according to population group. Representatives of the Roma community were recruited through word-of-mouth, community outreach, and invitations from support organizations. Service directors circulated information and consent forms via email to inform caregivers in child protection units. Parents and caregivers of children with disabilities were invited via social media and organisations that support them. The ethical procedures and data collection details are described elsewhere [10,26]. Prior to data collection, researchers outlined ground rules to ensure respectful and non-overlapping discussions. Interviews lasted approximately 60–75 min, and focus groups 60–90 min. Participants could pause, ask questions, or withdraw at any time; however, no withdrawals or procedural issues occurred. Audio recordings were securely stored on an encrypted drive accessible only to the research team. Ethical approval was granted by the Ethics Committee of Harokopio University (approval no. Γ-4081/06-10-2023), and the study adhered to relevant codes and regulations.

### 2.4. Data Collection Approach and Materials

A semi-structured approach was chosen to allow for more open-ended questions, providing flexibility to adjust or skip questions based on the conversation flow, thus facilitating a deeper exploration of relevant topics [28]. Three topic guides were developed by experts in childhood obesity and social research, each based on existing scientific knowledge and tailored to the target population: These were designed for parents of children with disabilities (Supplementary Material, Table S1), Roma community representatives (Supplementary Material, Table S2), and caregivers in child protection units (Supplementary Material, Table S3). Data collection was conducted using the same approach across all regions, with an equal number of interviews and focus groups in each region. The guides (Supplementary Material, Tables S1–S3) were piloted with a small number of individuals to ensure clarity, relevance, and comprehensiveness. Data saturation was assessed by the analysis team (T.B., V.S., and R.R.) in terms of both meaning saturation (analytic insight) and the emergence of new codes. After each interview or focus group, transcripts were reviewed against the evolving analytical framework. Recruitment continued until two consecutive interviews or focus groups yielded no new conceptual insights, defined as additional dimensions, interpretations, or relationships within or across existing themes, and no new codes, with findings only confirming or elaborating on prior results. Decisions regarding saturation were discussed during regular analytical meetings and documented in the shared drive meetings [31]. Data collection concluded once no new analytic insights and codes emerged across interviews and focus groups, following team consensus. To minimise bias, all researchers followed standardised procedures and received training from an experienced health psychologist and behavioral scientist prior to data collection. In this study, “engagement” refers to participants’ awareness of and interaction with obesity prevention policies and related initiatives.

### 2.5. Data Analysis

The audio recordings were transcribed verbatim, anonymized, and uploaded to NVivo software (version 14) for analysis. Thematic analysis was selected for its methodological flexibility and suitability for applied health research [23]. Three researchers (T.B., V.S., and R.R.) independently read all transcripts for familiarization. Coding was conducted line-by-line. Approximately twenty percent of the transcripts were independently double-coded by two researchers (T.B. and R.R.) to enhance analytic rigor and reliability. Coding discrepancies were discussed and resolved through consensus, with involvement of a third researcher (V.S.) where needed. This constituted researcher triangulation, defined as independent coding by multiple analysts followed by systematic comparison and consensus-building to strengthen credibility and minimize individual interpretive bias. Each participant group (Roma community representatives, caregivers in child protection units, and parents of children with disabilities) was initially analyzed separately to preserve group-specific perspectives. Subsequently, findings were compared and synthesized to identify shared themes and common patterns across groups. A combined inductive and deductive approach was applied. Themes were allowed to emerge from the data inductively while also mapping codes onto pre-identified thematic domains aligned with the research questions and study aims [23]. Codes were grouped into subthemes and overarching themes, and representative quotes were selected to illustrate key findings and shared patterns. Quotes were labelled consistently according to participant group. After reaching saturation, the final data were compiled in Excel, and the quotes were translated into English. Credibility and confidentiality procedures have been described elsewhere [26].

## 3. Results

### 3.1. Participant Characteristics

A total of seventy-two individuals participated in the study through individual interviews and focus groups. Forty-five parents of children with disabilities took part in individual interviews, equally represented in all three regions (fifteen participants per region). Among focus group participants: Twenty-one caregivers from child protection units participated in six focus groups (two per region), with focus group sizes ranging from three to five participants. In addition, six Roma community representatives participated in three focus groups (one per region), with focus group sizes ranging from one to four participants.

Table 1a,b describes the study design and coverage, and Table 1a,b describes the demographic characteristics of the study participants. Table S4 in the Supplementary Material presents the demographic characteristics of children with disabilities.

### 3.2. Themes

**Figure 1:**
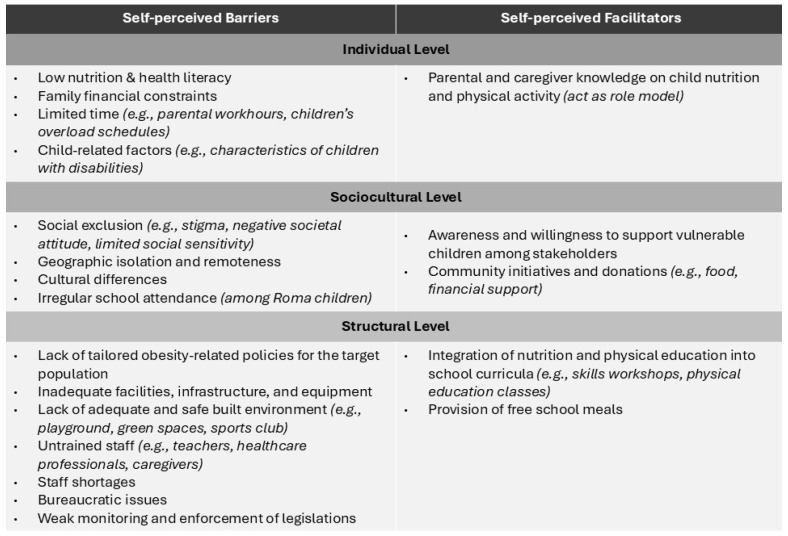
Key barriers and facilitators to engagement in obesity prevention policies among socially vulnerable children, based on participants’ views and experiences.

Four themes emerged from the thematic analysis, reflecting participants’ self-perceived barriers and facilitators to engagement in obesity-related prevention interventions. The first theme captures participants’ experiences engaging with policies addressing childhood obesity among socially vulnerable children in Greece. The remaining themes referred to individual, sociocultural, and structural factors that either facilitate or hinder their engagement. Despite their different backgrounds, similar barriers and facilitators were reported across all participant groups (caregivers of children in child protection units, Roma community representatives, and parents of children with disabilities). Figure 1 summarizes the key barriers and facilitators to engagement in obesity-related prevention interventions, as reported by the participants.

#### 3.2.1. Theme 1: Participants’ Experiences Related to Their Engagement in Policies Addressing Childhood Obesity in Socially Vulnerable Children in Greece

Nearly all participants reported some level of engagement with policies that promote healthy eating and physical activity for socially vulnerable children. The most frequently cited initiatives across all participant groups included physical education curricula, skills workshops, school-based healthy eating initiatives, financial aid programs, school meal provision, and community-led actions such as food donations. School canteen regulations were mentioned less frequently.

Caregivers most often referred to food donations and legislation governing food provision in childcare protection centers. Parents of children with disabilities primarily reported disability-related benefits and financial support for vulnerable families (e.g., the “market pass” voucher), and mentioned physical education curricula and adaptations for children with disabilities. Roma community representatives mentioned legislative measures less frequently than other groups. When they did mention them, these measures mainly concerned school meal provision and physical education curricula, while one focus group noted that children received municipal breakfast vouchers through partnerships with local bakeries.

#### 3.2.2. Theme 2: Self-Perceived Individual Barriers and Facilitators Influencing Engagement in Obesity Prevention Interventions

Parental health and nutrition literacy emerged as a key factor in shaping the ability of parents and caregivers to engage in obesity prevention interventions. Some parents and caregivers reported that being informed about nutritional issues supported healthier behaviors among their children through observation and imitation. On the other hand, about half of the parents, the majority of caregivers, and some Roma community representatives described having limited knowledge of the nutrition and physical activity needs of socially vulnerable children, which acted as a barrier to their engagement in obesity prevention efforts.

*“When new children arrive, episodes of overeating may occur due to anxiety about food availability or emotional difficulties. Caregivers notice this and try to manage overconsumption by offering healthier meals, until it decreases and the children feel more secure.”*

(Caregiver in child protection units, No. 213, Focus Group 2, Crete)

*“My child nor I were never taught anything about this (healthy nutrition), so we don’t really know […] If we knew, we could teach our children about nutrition, but since we don’t, they won’t learn either!”*

(Roma community Representative, No. 216, Focus Group, Crete)

Across all participant groups, family financial difficulties were frequently identified as a barrier to engaging in healthier lifestyle practices. A few parents emphasized the additional financial burden associated with therapy- and care-related expenses. Also, some parents and Roma community representatives reported that rising food prices, combined with existing financial strain, forced families to reduce the quantity and quality of certain food groups, such as olive oil, meat, and fish. This limited their ability to meet healthy eating recommendations.

*“When a family has a member with a disability, everyone is affected! These children have greater needs. The state should support these families. As long as the state does not provide support, the child inevitably will not adopt healthy eating habits!”*

(Parent of a child with disabilities, No. 317, Individual Interview, Attica)

In contrast, some parents noted that access to homegrown foods provided a valuable, cost-free food source that eased their financial burden.

*“Since we live in the countryside, we don’t always need to buy fruits and vegetables. There’s my grandfather’s garden and another grandfather’s orchard with oranges. It’s like a treasure trove here, so things are a bit easier because we have free access.”*

(Parent of a child with disabilities, No. 224, Individual Interview, Crete)

Some Roma community representatives cited long working hours that affect meal management. About half of parents of children with disabilities reported limited support when both parents are employed too. This reflects how their heavy workloads and exhaustion hinder their involvement in obesity-related policy interventions.

*"A family with a child with disabilities needs someone to be at home and not working all day for things to function properly. When parents are away all day, the children operate on autopilot because all the organization and contact is lost.”*

(Parent of a child with disabilities, No. 117, Individual Interview, Thessaly)

Moreover, some parents perceived that children with disabilities were rarely physically active, because demanding weekly schedules limited opportunities to participate in extracurricular activities.

*“He mostly goes to the park on weekends because his weekly schedule is very demanding. He’s not very active daily due to the intense program related to his spectrum condition.”*

(Parent of a child with disabilities, No. 114, Individual Interview, Thessaly)

Furthermore, children’s specific needs and characteristics were widely perceived as significant barriers to full engagement in obesity-related prevention interventions. In this instance, most parents highlighted that children with disabilities often experience feeding challenges, including selective eating, food preferences, and chewing difficulties. They also noted that these children have difficulty participating in the same physical activities as typically developing peers.

*"There are no organized activities for typical children who accept children with autism. They said he must attend a special association. At a typical school, he misses half of the physical education classes because he cannot keep up. So, how can he be physically active?"*

(Parent of a child with disabilities, No. 311, Individual Interview, Attica)

#### 3.2.3. Theme 3: Self-Perceived Sociocultural Barriers and Facilitators Influencing Engagement in Obesity Prevention Interventions

Almost half of the participants emphasized that awareness, willingness, and active support from stakeholders, including teachers, healthcare professionals, and caregivers, facilitated engagement in obesity prevention interventions and the adoption of healthier nutrition and physical activity practices.

*“The physical education teacher has made sure to adapt the activities during physical education classes so that the child can participate, either by doing exercises with different equipment, such as hoops, balls, and other materials.”*

(Parent of a child with disabilities, No. 234, Individual Interview, Crete)

*“Great importance is placed on ensuring children have proper meals, not only by us here in the management, but also by the caregivers. They are very focused on food and on making sure the children eat well.”*

(Caregiver in child protection units, No. 212, Focus Group 2, Crete)

Social exclusion was widely reported as a sociocultural barrier, particularly by almost all Roma community representatives and some parents. Parents emphasized that low public awareness and poor respect for disability infrastructure further limited engagement opportunities for them and their children, reinforcing experiences of exclusion. Some Roma community representatives observed that Roma children attending mixed schools had better access to resources and activities than their peers in Roma community schools. This was perceived as limiting community engagement and fostering social inequalities.

*“Some children attend schools exclusively for Roma, while others go to mixed schools. The situation is better in mixed schools because they have more resources and more activities. Even physical education is different. They have more balls, better equipment, and so on.”*

(Roma community representative, No 324, Focus Group, Attica)

Moreover, half of the parents and some caregivers emphasized the negative societal attitudes. Parents believed that societal stigma limited their children’s socialization and participation in group activities. Similarly, one caregiver noted a lack of social sensitivity toward children in child protection units, underscoring the need for interventions that promote awareness, inclusion, and respect for different living situations.

*“I think that school staff should be informed about children living in institutions—about their living conditions and the difficulties they’ve faced. This would help raise awareness among students and help them understand these challenges [...] Not just for children from institutions, but also those from different countries, or with disabilities. We talk about inclusion and inclusive spaces, but this still hasn’t been implemented!"*

(Caregiver in child protection units, No 307, Focus Group 1, Attica)

Cultural differences were also reported to hinder equal participation in obesity-related interventions, particularly by most Roma community representatives. They emphasized that their cultural characteristics are often overlooked and emphasized the need for empathetic, tailored approaches to ensure full inclusion. Additionally, some Roma representatives highlighted irregular school attendance as a major barrier to children’s, and by extension their families’ and communities’, participation in obesity-related interventions.

*“Some Roma children do not attend school. This is a problem we face too! Since they are not in school, they miss out on learning important things. They are not at home either; they spend all their time outside.”*

(Roma community representative, No. 324, Focus Group, Attica)

In contrast, the majority of caregivers and Roma community representatives identified community-led initiatives as important facilitators, encouraging their involvement in obesity prevention strategies. Some Roma representatives highlighted local community support through donations for disadvantaged Roma families. Additionally, most caregivers cited the positive impact of food donations on the nutrition of children living in child protection units. Some added that local sports clubs often accept these children without membership fees.

*“Each academy provides support, and we can enroll children without a membership fee. The municipality also offers programs, including a dance section and cultural activities, where we can take our children for free.”*

(Caregiver in child protection units, No. 306, Focus Group 1, Attica)

Geographical factors were also identified, particularly by most parents, who described long distances to therapy centers and organized physical activities, as limiting access and contributing to more sedentary lifestyles, including increased screen time. Despite such challenges, some parents reported being strongly motivated to support their children and traveled long distances to enable participation.

*“Regarding physical activity, there is nothing available here (small town in Crete). When we lived in […] (the name of a big Cretan city), my son participated in theater, music, and crafts. Here, everything is cut off! He used to play boccia, but we lost access to it after moving here. Now that there are no activities available, he spends his afternoons on the computer.”*

(Parent of a child with disabilities, No. 205, Individual Interview, Crete)

*“My child does therapeutic horseback riding twice a week, and the travel is worth it! It’s truly life-saving!! It helps that my child has found something he enjoys and wants to do, so it doesn’t feel like a struggle at all!”*

(Parent of a child with disabilities, No. 310, Individual Interview, Attica)

#### 3.2.4. Theme 4: Self-Perceived Structural Barriers and Facilitators Influencing Engagement in Obesity Prevention Interventions

Participants across groups commonly viewed the school environment as an important setting for promoting healthy behaviors among children and could encourage their engagement, as well as encourage the engagement of their families and communities. However, a few participants perceived that the school did not contribute to this, while as one caregiver mentioned:

*“No child from the facility has ever come to tell us that someone visited today and talked to them about this topic. And that really surprised me, because they come and talk to them about everything else.”*

(Caregiver in child protection units, No. 210, Focus Group 1, Crete)

Almost half of the participants noted that initiatives, such as “skills workshops” and physical education, were particularly effective in enhancing participation and fostering inclusion. School meal provision was also widely reported as fundamental to children’s engagement in healthy eating practices, especially for low-income families, as it both promoted healthy dietary habits and ensured access to nutritious food.

*“There is a program in a school providing meals twice a week, with a rotation of legumes, fish, and meat. Beyond the quality of the food, each meal includes a salad or fruit, providing children with a whole meal. This helps them understand what a proper lunch and mid-morning snack should be like.”*

(Caregiver in child protection units, No. 211, Focus Group 1, Crete)

Despite acknowledging the importance of schools, key obstacles arose across all participant groups, including weak monitoring mechanisms of policies. For example, in one focus group, caregivers noted that the quality of school meals lacked proper evaluation mechanisms, while some parents and caregivers noted that school canteens continued to sell prohibited foods, undermining healthy eating efforts. In addition, some participants across all groups mentioned that the implementation of physical education was inconsistent and often dependent on individual teachers’ discretion, which limited equitable access to physical activity for vulnerable children.

*“Sometimes it’s like, ‘You don’t want to do physical education? You don’t want to run? Then stay out and sit in the corner.’ This happens everywhere. ‘Oh, you didn’t put on your uniform? Fine, it’s ok, just stay in the corner.’”*

(Roma community representative, No. 132, Focus Group, Thessaly)

Beyond school settings, some parents and caregivers perceived bureaucratic challenges, including difficulties in scheduling appointments with doctors at public hospitals and excessive administrative procedures, as barriers to their involvement in health policy interventions and the promotion of well-being among vulnerable children.

*“We can’t currently receive the celiac disease allowance. Currently, I have to book an appointment at the hospital, but I can’t get one, because they’re always full. So, I just leave it!”*

(Parent of a child with disabilities, No. 222, Individual Interview, Crete)

Structural limitations related to human resources and staff availability were frequently reported by all participant groups, including schools, child protection units, and communities. For instance, most of the parents noted delays in hiring educational staff, combined with a lack of special education teachers, in both typical and special education schools, which negatively affected inclusion in initiatives promoting healthy nutrition and physical activities. Additionally, the limited time allocated to physical education further restricted opportunities for regular physical activity, as some parents noted.

*"How can the school help? Especially speaking from my background as a teacher, physical education classes that used to be two or three hours a week have been reduced to just one hour in some grades?"*

(Parent of a child with disabilities, No. 119, Individual Interview, Thessaly)

*“The physical education teacher has no training for special needs and does not know how to handle these children! The aide also does not know what to do during physical education, so they take a break and leave the child in their wheelchair, on the side of the schoolyard!”*

(Parent of a child with disabilities, No. 328, Individual Interview, Attica)

Moreover, as some parents noted, the lack of trained physical education teachers and limited awareness of the needs of children with disabilities often led to their exclusion from physical education classes. Parents also expressed hesitation in trusting untrained staff, which further limited their children’s participation. On the other hand, some caregivers reported that training in childhood nutrition, which was the institution’s initiative, improved their knowledge and everyday practices, such as reducing the use of food as a reward, and underscoring the importance of staff training in child protection units.

*“Before the nutrition training, it was common for caregivers to reward children with food, but now they manage it differently.”*

(Caregiver in child protection units, No. 214, Focus Group 2, Crete)

According to most participants, nutrition or physical activity interventions specifically targeting vulnerable children were rare, and when present, these interventions were mostly implemented in schools. The majority of participants from each group stated they have never received official training or information on healthy eating and physical activity guidelines for children, nor participated in related initiatives.

*“I believe the only role the regional authority plays regarding the meal plan is to check whether we have one. And even that depends on the regional representative who happens to come and whether they want to check the meals we serve. But that’s it, nothing more.”*

(Caregiver in child protection units, No. 321, Focus Group 2, Attica)

Some participants also emphasized the limited public and social support available to families and stakeholders caring for socially vulnerable children. Several caregivers described the lack of structural support within the child protection center, while a few parents noted that no social protection professionals had ever provided guidance or assistance regarding their child’s special needs.

*“No social worker, psychologist, or psychiatrist ever came to see us and ask, ‘How are you? How are you managing all this?”*

(Parent of a child with disabilities, No. 111, Individual Interview, Thessaly)

Structural limitations related to infrastructure and equipment were frequently reported across schools, child protection units, and communities. Most parents emphasized insufficient facilities and equipment for children with disabilities in both regular and special education schools. Additionally, nearly half of the caregivers highlighted limited infrastructure in child protection units, while one Roma focus group stressed the absence of basic provisions in the community, such as electricity, which severely impacted the eating habits and overall health of Roma children. Conversely, some caregivers and parents noted that equipment and facilities, when available, encouraged a more active and inclusive daily lifestyle for children.

*"No child, or parent, can focus on preventing childhood obesity, or even see it as important, when they do not have a home, electricity, running water, or clothes for their children!”*

(Roma community representative, No. 219, Focus Group, Crete)

An accessible and supportive surrounding environment, including sports facilities, green spaces, playgrounds, and disability-friendly infrastructure such as seaside trails, was considered essential for encouraging regular physical activity among socially vulnerable children, as highlighted by several parents and caregivers.

*“The municipality installed a seaside track for a person with special needs in our neighborhood, which is a seaside area. This makes my son independent!”*

(Parent of a child with disabilities, No. 205, Individual Interview, Attica)

*“We are lucky, because most of the playgrounds are within close distance for the children, so we try—if not every day, then whenever there is availability and a gap in their schedule—to take them outside.”*

(Caregiver in child protection units, No. 323, Focus Group 2, Attica)

However, the majority of participants reported the absence of such supportive infrastructure in their neighborhoods. This perception was particularly pronounced among Roma participants. Nearly all Roma community representatives exclusively highlighted the lack of an enabling environment in their communities, as well as the widespread availability of calorie-dense ultra-processed foods, which further discourages healthy eating habits among Roma children.

*“A Roma kid will go to the grocery store, and even if they have no money, they tell the shopkeeper, ‘Give me a croissant, some crisps, a juice, anything! My father will pay you later’ and they will give it to them.”*

(Roma community representative, No. 132, Focus Group, Thessaly)

*“The playground is completely unsafe! There is gravel on the ground, so you can get hurt. It is also very dirty. They have installed one slide that is for children over seven or eight years old. Overall, it is very poorly done, as I believe is the case with most playgrounds.”*

(Parent of a child with disabilities, No. 120, Individual Interview, Thessaly)

Limited and fragmented funding was identified as a significant structural barrier that affected vulnerable children’s equal participation in policies promoting healthy eating and physical activity. The majority of caregivers reported that underfunding of child protection units restricted the provision of nutritious food and, in some cases, participation in organized physical activities among institutionalized children. Most parents emphasized that disability-related benefits primarily covered therapy-related costs and were often insufficient to address broader needs related to healthy eating and physical activity.

*“The uniforms for organized physical activities, for example, are something we have to cover ourselves! Every year, each team wants its own uniform. They want two uniforms: one for training and one for matches.”*

(Caregiver in child protection units, No. 308, Focus Group 1, Attica)

*“There is financial support from the state, although it is insufficient. It covers therapy costs to some extent, but we have to make up the rest ourselves!”*

(Parent of a child with disabilities, No. 314, Individual Interview, Attica)

## 4. Discussion

This is the first comprehensive qualitative study exploring the experiences and self-perceived factors that influence engagement in obesity prevention strategies among parents, caregivers, and community representatives who support socially vulnerable children in Greece. By integrating the perspectives of parents of children with disabilities, Roma community representatives, and caregivers in child protection units from various geographical areas, the study provides new insights into how individual, sociocultural, and structural factors influence engagement in obesity-related interventions.

Participants’ experiences with childhood obesity–related interventions mainly highlighted policies implemented in schools, including skills workshops, physical education, and healthy eating initiatives. These initiatives emphasize the central role of school-based programs in creating environments that positively influence children’s nutritional and physical activity behaviors, as shown in previous research [32,33,34]. Additionally, many participants reported receiving government financial support, which was often inadequate to cover nutrition-related expenses. Previous research indicates that social protection measures, including adequate funding and financial assistance, can improve child well-being by enhancing access to food, health services, and other basic necessities, particularly among socially vulnerable families [35,36,37].

Overall, the determinants influencing engagement were largely similar among participants in this study. These findings reflect common experiences and perceptions across socially vulnerable populations, suggesting the presence of underlying shared needs that should be considered collectively. At the same time, some group-specific challenges were also evident as well. Parents of children with disabilities primarily reported inadequate infrastructure and untrained professionals in education and healthcare services. Roma community representatives highlighted language barriers and the absence of culturally tailored approaches. Caregivers in child protection units primarily pointed to insufficient funding and limited prioritization of resources related to children’s nutrition and organized physical activity.

At the individual level, participants most frequently identified low parental health and nutrition literacy, and family financial constraints as key barriers to their engagement in obesity prevention. Low nutrition literacy, influenced by limited knowledge, language barriers, and inadequate access to tailored information, has been consistently linked to lower participation in health interventions among socially vulnerable populations [12,34,38,39]. Financial difficulties, including rising food costs, often lead socially vulnerable families to choose cheaper, less nutritious options, as previous findings have shown [4,25,40]. Roma families often experience limited access to adequate health services due to severe poverty [12,39]. Moreover, families of children with disabilities described additional care-related expenses as a significant burden, which is consistent with previous findings [10,27]. Notably, children were more likely to adopt healthier habits when participants modeled healthy behaviors [10,27,41]. These findings highlight the impact of low health and nutrition literacy, as well as family financial constraints, on engagement in obesity prevention among children at risk of poverty and social exclusion, underscoring the need for affordable and accessible interventions.

Additionally, participants’ limited time due to demanding work schedules, caregiving responsibilities, and children’s overloaded routines further constrained engagement in healthy eating and physical activity. According to research, busy schedules often lead parents to rely on fast or processed meals instead of homemade and nutritious options [40], while children’s overloaded schedules restrict their physical activity [27,41,42].

Sociocultural factors influenced engagement through multiple pathways. Social exclusion and negative societal attitudes, such as stigma, were highlighted as key barriers by the study participants, limiting socialization and participation in nutrition and physical activity policy interventions [12,17,38,43]. Parents of children with disabilities also reported low public awareness, poor respect for disability infrastructure, and geographic isolation, including long distances. These factors contribute to further social exclusion and limit participation in physical activity and therapies for these children, as cited previously [27,41]. Furthermore, cultural differences, particularly within Roma communities, were reported to hinder engagement. Ethnic, linguistic, and religious diversity is often overlooked, resulting in interventions that are not tailored to their needs, highlighting the importance of empathetic and culturally sensitive approaches [12,38,39]. Irregular school attendance was also noted as an obstacle, with prior research emphasizing that improving education quality and reducing dropout rates enhances health education, trust, inclusion, and intervention effectiveness among Roma children [12,26,39]. These findings underscore the need to tailor policies to the specific needs of at-risk children, considering linguistic and cultural differences, as well as social exclusion and geographic barriers, to promote equity and inclusion [7,34,44,45].

Participants identified the following sociocultural facilitator: awareness and motivation of stakeholders working directly with children at risk of poverty and social exclusion, such as teachers, healthcare professionals, and caregivers in child protection units. This facilitator was important for creating supportive nutritional environments in residential settings and schools, similar to other findings [13,17,33,46]. Additionally, community-led initiatives and food donations, particularly in child protection units, provided essential support as discussed, though earlier studies have shown that reliance on these resources may limit dietary diversity and compromise the nutritional quality of food for institutionalized children [47,48].

At a structural level, participants reported that the existence of nutrition and physical activity policy interventions, mainly in schools, facilitated engagement in obesity prevention interventions. This was particularly true for measures such as free school meals, which support healthy eating, provide essential nutrition for families facing financial difficulties, and reduce stigmatization [37,46,49]. Despite these facilitators, participants consistently highlighted the need for policy interventions tailored to the specific needs of socially vulnerable children. Insufficient human resources, including staff shortages and limited trained personnel, emerged as a major structural barrier to equitable engagement in obesity prevention. According to the study participants, limited trained personnel both undermined parental trust and excluded children with disabilities from physical activity. This reinforces social exclusion and limits equity, negatively affecting the prevention of childhood obesity for socially vulnerable children [37,50,51]. In contrast, trained and qualified staff facilitated engagement in nutrition and physical activity interventions, consistent with previous findings [37]. Limited government-led training and support within the social protection system further contributed to inconsistent service delivery for disadvantaged children, similar to other research [13,25,41,42,52].

Study participants also reported persistent barriers related to limited access to safe and suitable surrounding environments that support regular physical activity among vulnerable children, including sports facilities, green spaces, playgrounds, and disability-friendly infrastructure. The lack of these environments restricts participation and equity, discouraging regular physical activity among vulnerable children [7,41,42,43]. Roma community representatives uniquely described inadequate basic amenities such as electricity, alongside widespread availability of calorie-dense, ultra-processed foods, reinforcing obesogenic environments within Roma communities, consistent with previous studies [39,53]. Furthermore, weak government monitoring mechanisms, combined with complex bureaucratic procedures, further hindered comprehensive participation, as evidenced previously [6,11,25,37]. Strengthening inclusive infrastructure, improving regulatory monitoring, and simplifying administrative procedures are essential to reducing structural barriers and promoting equitable participation in obesity prevention interventions [7,20,27,44]. Finally, insufficient funding for vulnerable families and childcare institutions reinforces structural inequalities and reduces access to quality services and consistent nutrition [13,26,54].

This qualitative study has several strengths. The combination of inductive and deductive thematic analysis provided both data-driven and theory-informed insights, offering a comprehensive understanding of the barriers and facilitators that influence participation and engagement in obesity prevention policies and activities for socially vulnerable children in Greece. This approach provided important context for the topic, revealing perspectives that are often overlooked by quantitative methods. Semi-structured interview guides enabled a detailed exploration of factors influencing participants’ engagement in obesity prevention policies for children from disadvantaged settings, with data saturation indicating that the sample size was sufficient to capture meaningful themes [23]. Additionally, using both focus groups and interviews increased the degree of detailed information. Focus groups facilitated rich data collection, and interviews ensured confidentiality and promoted open discussion [28,30]. To address challenging group dynamics, the researchers established clear ground rules prior to conducting the focus groups. An additional strength was the inclusion of participant groups representing a substantial number of children from disadvantaged settings across three different national regions. This enhanced the robustness and potential generalizability of the findings to socially vulnerable children in other regions of the country. However, due to the specific nature of the study, its generalizability is limited to different populations and settings.

One study limitation was the low participation in the Roma focus groups, with only six participants. Additionally, Roma community representatives were included instead of parents in order to capture broader community perspectives, even though most Roma were also parents. These factors may limit the generalizability of the findings to Roma communities in Greece. These factors also reflect the low community readiness and limited awareness of nutrition and obesity issues reported in previous research [12]. Nevertheless, Roma community representatives, like the other participant groups, were willing to share their experiences and perceptions. Similarly, parents of children with disabilities and caregivers in child protection units may have provided responses influenced by caregiving demands or group dynamics, potentially shaping the depth and focus of the data. Additionally, qualitative research can be prone to information bias because data quality depends on the interviewer’s ability to elicit responses. To mitigate this risk, team members underwent training with an experienced health psychologist and behavioral scientist, ensuring consistency, rigor, and inter-rater reliability in data collection. Nevertheless, cultural and contextual factors may still have influenced participants’ responses, particularly in focus group discussions. Additionally, some relevant contextual aspects may not have been explored either because they were beyond the study’s scope or because participants did not raise them, leaving their influence uncertain. Finally, this study may not have fully captured the perspectives of the Roma community due to low participation and reliance on community representatives, which could have influenced the identified barrier profile and led to an incomplete understanding of their specific challenges. Future research should adopt targeted and inclusive recruitment strategies to ensure broader representation of these populations.

## 5. Conclusions

Engagement in obesity prevention policies targeting socially vulnerable children is shaped by multiple, interrelated determinants across individual, sociocultural, and structural levels. Translating these findings into practice requires targeted and coordinated action. Prioritized barriers and corresponding actionable levers could include:

- Inadequate school food environments: Strengthening the implementation and monitoring of free school meal programs and enhancing compliance checks of school canteens (Implementers: Government through ministry involvement, local authorities, school administration).
- Limited time and opportunities for physical activity: Establishing and enforcing a minimum number of hours for physical education within the national curriculum, and supporting structured extracurricular physical activity programs (Implementers: Government through ministry involvement, school administrations).
- Unsafe or inaccessible built environments: Improving safety, maintenance, and accessibility of green spaces and community sports facilities, particularly in underserved areas such as rural or low SES areas (Implementers: Municipalities, regional authorities, government through ministry involvement).
- Financial constraints: Provision of targeted financial support or subsidies to socially vulnerable groups to facilitate access to nutritious food and organized physical activity (Implementers: government through ministry involvement, social welfare agencies).
- Cultural and linguistic barriers to engagement: Developing and deliveing culturally and linguistically tailored obesity prevention interventions, including interpreter support and collaboration with community mediators (Implementers: government through ministry involvement, local health authorities, community organizations).
- Limited health literacy and training capacity: Offering structured training programs for educators, caregivers, and parents who support socially vulnerable children, focusing on nutrition, physical activity, and health literacy (Implementers: government through ministry involvement).

Embedding such actions within a coordinated, multisectoral framework may enhance the effectiveness and equity of obesity prevention efforts targeting children at risk of poverty and social exclusion.

## Figures and Tables

**Table 1:** (**a**) Study design and coverage. (**b**) Demographic characteristics of the study participants.

(**a**)			
	**Caregivers in Child Protection Units**	**Roma Community Representatives**	**Parents of Children with Disabilities**
Data collection method	Focus Group	Focus Group	Individual interviews
Number of focus groups per region (*n*)	2	1	-
Number of participants (*n*)	21	6	45
Area of residence (*n*)			
Attica	6	1	15
Thessaly	8	1	15
Crete	7	4	15
(**b**)			
		Study Participants (N = 72)	
Age (years) Median (IQR) *			
Caregivers in Child Protection Units		42 (33, 50)	
Roma community representatives		25 (22, 28)	
Parents of children with disabilities		44 (41, 51)	
Gender *n* (%)			
Men		10 (14%)	
Women		62 (86%)	
Level of education *n* (%)			
Postgraduate studies		11 (15%)	
Undergraduate studies		29 (40%)	
Post-secondary education		15 (22%)	
High School		16 (22%)	
Junior High School		1 (1%)	
Employment *n* (%)			
Full-time		51 (71%)	
Part-time		4 (5%)	
Unemployment		17 (24%)	
Parenthood n (%)			
Yes		61 (85%)	
No		11 (15%)	

* IQR—with interquartile range.

## Data Availability

The data presented in this study are not available due to privacy.

## Supplementary Materials

The following supporting information can be downloaded at: https://www.mdpi.com/article/10.3390/healthcare14050620/s1, Tables S1–S3: Respective topic guides for conducting semi-structured interviews with participants. (parents of children with disabilities, Roma community representatives, and caregivers in child protection units); Table S4: Characteristics of children with disabilities.

## References

[1] Mah J.C., Penwarden J.L., Pott H., Theou O., Andrew M.K. (2023). Social vulnerability indices: A scoping review. BMC Public Health.

[2] (2022). WHO WHO Guidance on Research Methods for Health Emergency and Disaster Risk Management. https://iris.who.int/handle/10665/363502.

[3] European Commission (2021). European Child Guarantee.

[4] Papamichael M.M., Karatzi K., Mavrogianni C., Cardon G., De Vylder F., Iotova V., Usheva N., Tankova T., González-Gil E.M., Kivelä J. (2022). Socioeconomic vulnerabilities and food intake in European children: The Feel4Diabetes Study. Nutrition.

[5] Iguacel I., Fernández-Alvira J.M., Bammann K., De Clercq B., Eiben G., Gwozdz W., Molnar D., Pala V., Papoutsou S., Russo P. (2016). Associations between social vulnerabilities and dietary patterns in European children: The Identification and prevention of Dietary- and lifestyle-induced health EFfects in Children and infantS (IDEFICS) study. Br. J. Nutr..

[6] Dana L.M., Ramos-García C., Kerr D.A., Fry J.M., Temple J., Pollard C.M. (2025). Social Vulnerability and Child Food Insecurity in Developed Countries: A Systematic Review. Adv. Nutr..

[7] Iguacel I., Gasch-Gallén Á., Ayala-Marín A.M., De Miguel-Etayo P., Moreno L.A. (2021). Social vulnerabilities as risk factor of childhood obesity development and their role in prevention programs. Int. J. Obes..

[8] Ayala-Marín A.M., Iguacel I., Miguel-Etayo P.D., Moreno L.A. (2020). Consideration of Social Disadvantages for Understanding and Preventing Obesity in Children. Front. Public Health.

[9] Mannino A., Halilagic A., Argyropoulou M., Siopis G., Roussos R., Svolos V., Mavrogianni C., Androutsos O., Mouratidou T., Manios Y. (2024). The Role of Energy Balance-Related Behaviors (EBRBs) and Their Determinants on the Prevalence of Overweight and Obesity in Children in Need, in Greece: A Scoping Review.

[10] Svolos V., Strongylou D.E., Argyropoulou M., Stamathioudaki A.M., Michailidou N., Balafouti T., Roussos R., Mavrogianni C., Mannino A., Moschonis G. (2025). Parental Perceptions About Energy Balance Related Behaviors and Their Determinants Among Children and Adolescents Living with Disability: A Qualitative Study in Greece. Healthcare.

[11] Cyril S., Green J., Nicholson J.M., Agho K., Renzaho A.M.N. (2016). Exploring Service Providers’ Perspectives in Improving Childhood Obesity Prevention Among CALD Communities in Victoria, Australia. PloS ONE.

[12] Islam S., Small N., Bryant M., Yang T., Cronin de Chavez A., Saville F., Dickerson J. (2019). Addressing obesity in Roma communities: A community readiness approach. Int. J. Hum. Rights Healthcare.

[13] DeLacey E., Tann C., Groce N., Kett M., Quiring M., Bergman E., Garcia C., Kerac M. (2020). The nutritional status of children living within institutionalized care: A systematic review. PeerJ.

[14] Manios Y., Moschonis G., Androutsos O. (2022). Obesity and Related Cardiometabolic Diseases. Dianeosis Rep..

[15] Manios Y., Androutsos O., Katsarou C., Vampouli E.A., Kulaga Z., Gurzkowska B., Iotova V., Usheva N., Cardon G., Koletzko B. (2018). Prevalence and sociodemographic correlates of overweight and obesity in a large Pan-European cohort of preschool children and their families: The ToyBox-study. Nutrition.

[16] UNICEF (2023). Launch of the National Action Against Childhood Obesity in Greece 2023–2025.

[17] Markert J., Herke M., Bartels A., Gosse K., Roick J., Herz-Jakoby A., Täubig V., Schröer W., Richter M. (2021). Food practices and nutrition of children and adolescents in residential care: A scoping review. Appetite.

[18] Hellenic Statistical Authority (2021). Health Survey 2019: Health of Children 2–14 Years Old. https://www.statistics.gr/en/statistics/-/publication/SHE22/-.

[19] Eurostat (2025). 13.6% of EU Children Faced Material Deprivation in 2024—News Articles—Eurostat. https://ec.europa.eu/eurostat/web/products-eurostat-news/w/ddn-20250613-2?utm_source=chatgpt.com.

[20] Balafouti T., Roussos R., Argyropoulou M., Svolos V., Mavrogianni C., Mannino A., Moschonis G., Androutsos O., Manios Y., Mouratidou T. (2025). A Policy-Driven Scoping Review of the Regulatory and Operational Framework Addressing Obesity in Children in Need in Greece. Child. Soc..

[21] WHO (2016). Report of the Commission on Ending Childhood Obesity.

[22] UNICEF (2020). Nutrition, for Every Child: UNICEF Nutrition Strategy 2020–2030.

[23] Clarke V., Braun V. (2013). Successful Qualitative Research: A Practical Guide for Beginners.

[24] Mohammadpour-Ahranjani B., Pallan M.J., Rashidi A., Adab P. (2014). Contributors to childhood obesity in Iran: The views of parents and school staff. Public Health.

[25] Almutairi S.H., Alhamidi S.A. (2024). Exploring parents’ perceptions and experiences of childhood obesity and management in Riyadh, Saudi Arabia: An interpretive qualitative study. BMC Public Health.

[26] Balafouti T., Strongylou D.E., Svolos V., Argyropoulou M., Roussos R., Mavrogianni C., Manidis A., Halilagic A., Moschonis G., Androutsos O. (2025). Addressing Childhood Obesity in Children in Need in Greece: Policy Implementers’ Knowledge, Perceptions and Lessons for Effective Implementation. Nutrients.

[27] Shields N., Synnot A. (2016). Perceived barriers and facilitators to participation in physical activity for children with disability: A qualitative study. BMC Pediatr..

[28] Bryman A. (2016). Social Research Methods.

[29] Council of the European Union (2021). Council Recommendation (EU) 2021/1004 of 14 June 2021 Establishing a European Child Guarantee. https://eur-lex.europa.eu/legal-content/EN/TXT/?uri=uriserv%3AOJ.L_.2021.223.01.0014.01.ENG&toc=OJ%3AL%3A2021%3A223%3ATOC.

[30] Ahmed S.K., Mohammed R.A., Nashwan A.J., Ibrahim R.H., Abdalla A.Q., Ameen B.M.M., Khdhir R.M. (2025). Using thematic analysis in qualitative research. J. Med. Surgery Public Health.

[31] Hennink M., Kaiser B.N. (2022). Sample sizes for saturation in qualitative research: A systematic review of empirical tests. Soc. Sci. Med..

[32] Khambalia A.Z., Dickinson S., Hardy L.L., Gill T., Baur L.A. (2012). A synthesis of existing systematic reviews and meta-analyses of school-based behavioural interventions for controlling and preventing obesity. Obes. Rev..

[33] Lambrinou C.P., Androutsos O., Karaglani E., Cardon G., Huys N., Wikström K., Kivelä J., Ko W., Karuranga E., Tsochev K. (2020). Effective strategies for childhood obesity prevention via school based, family involved interventions: A critical review for the development of the Feel4Diabetes-study school based component. BMC Endocr. Disord..

[34] Halilagic A., Roussos R., Argyropoulou M., Svolos V., Mavrogianni C., Androutsos O., Mouratidou T., Manios Y., Moschonis G. (2024). Interventions to promote healthy nutrition and lifestyle and to tackle overweight and obesity amongst children in need in Europe: A rapid literature review. Front. Nutr..

[35] Aurino E., Guinti S. (2022). Social Protection for Child Development in Crisis: A Review of Evidence and Knowledge Gaps. World Bank Res. Obs..

[36] Reeves A., Rachel L., Valerie T. (2021). Family policy and food insecurity: An observational analysis in 142 countries. Lancet Planet. Health.

[37] Saleh M., Ba-Break M., Abahussin A. (2024). Barriers and facilitators of school-based obesity prevention interventions: A qualitative study from the perspectives of primary school headteachers. J. Health Popul. Nutr..

[38] Cyril S., Nicholson J.M., Agho K., Polonsky M., Renzaho A.M. (2017). Barriers and facilitators to childhood obesity prevention among culturally and linguistically diverse (CALD) communities in Victoria, Australia. Aust. N. Z. J. Public Health.

[39] Rechel B., Blackburn C.M., Spencer N.J., Rechel B. (2009). Access to health care for Roma children in central and eastern Europe: Findings from a qualitative study in Bulgaria. Int. J. Equity Health.

[40] Ravikumar D., Spyreli E., Woodside J., McKinley M., Kelly C. (2022). Parental perceptions of the food environment and their influence on food decisions among low-income families: A rapid review of qualitative evidence. BMC Public Health.

[41] McGarty A.M., Melville C.A. (2018). Parental perceptions of facilitators and barriers to physical activity for children with intellectual disabilities: A mixed methods systematic review. Res. Dev. Disabil..

[42] Reinehr T., Dobe M., Winkel K., Schaefer A., Hoffmann D. (2010). Obesity in Disabled Children and Adolescents An Overlooked Group of Patients. Dtsch. Arzteblatt..

[43] Law M., Petrenchik T., King G., Hurley P. (2007). Perceived Environmental Barriers to Recreational, Community, and School Participation for Children and Youth With Physical Disabilities. Arch Phys. Med. Rehabil..

[44] Coupe N., Cotterill S., Peters S. (2018). Tailoring lifestyle interventions to low socio-economic populations: A qualitative study. BMC Public Health.

[45] Park J., Ten Hoor G., Cho J., Won S., Ryu S., Lau S.T. (2024). Obesity-related behaviors and health-related quality of life in socioeconomically vulnerable children: A cross-sectional study. J. Pediatr. Nurs..

[46] Dalma A., Zota D., Kouvari M., Kastorini C.M., Veloudaki A., Ellis-Montalban P., Petralias A., Linos A., DIATROFI Program Research Team (2018). Daily distribution of free healthy school meals or food-voucher intervention? Perceptions and attitudes of parents and educators. Appetite.

[47] Umar A., Anigo K., Nwajagu I. (2021). Assessment of Anthropometric Indices and Micronutrient Status of Children under Five in Orphanages of Kaduna Metropolis. Am. J. Food Nutr..

[48] Farid M.F., Rehman A., Khaliq A.M., Ali N., Tareq A.H. (2024). Malnutrition and associated risk factors in orphanages in Punjab, Pakistan: An analytical study. BMJ Nutr. Prev. Health.

[49] Meshkovska B., Gebremariam M.K., Atukunda P., Iversen P.O., Wandel M., Lien N. (2023). Barriers and facilitators to implementation of nutrition-related actions in school settings in low- and middle-income countries (LMICs): A qualitative systematic review using the Consolidated Framework for Implementation Research (CFIR). Implement Sci. Commun..

[50] World Obesity Federation (2021). Addressing Childhood Obesity Through City-Level Interventions. http://www.worldobesity.org.

[51] Kayitesi C., Tagoe N., Acheampong P.R. (2025). The role of teachers in the prevention and management of childhood obesity among school-aged children in Ghana: A cross-sectional study. Front. Public Health.

[52] Finkelstein D.M., Petersen D.M., Schottenfeld L.S. (2017). Promoting children’s physical activity in low-incomecommunities in Colorado: What are the barriers and opportunities?. Prev. Chronic Dis..

[53] Loring B., Robertson A. (2014). Obesity and inequities: Guidance for addressing inequities in overweight and obesity. World Health Organ Eur..

[54] Ng M., Fleming T., Robinson M., Thomson B., Graetz N., Margono C., Mullany E.C., Biryukov S., Abbafati C., Abera S.F. (2014). Global, regional, and national prevalence of overweight and obesity in children and adults during 1980–2013: A systematic analysis for the Global Burden of Disease Study 2013. Lancet.

