# Supplementary material for: Barriers and Facilitators to Increased Parental, Caregiver, and Community Engagement in Obesity Prevention Targeting Vulnerable Children: A Qualitative Study in Greece
Source: Healthcare (Basel). 2026 Feb 28;14(5):620. doi: 10.3390/healthcare14050620 (PMC12984798; doi:10.3390/healthcare14050620)
Supplement: Supplementary file 1 [file healthcare-14-00620-s001.zip › healthcare-4019882-supplementary.pdf]

Supplementary Material

Tables S1-S3. These tables present respective topic guides for conducting semi-structured interviews and focus groups with study participants.

Table S4. The table provides information on the characteristics of children with disabilities.

Table S1. Topic guides for conducting semi-structured interviews with parents of children with disabilities.

1.	Would you like to tell me about the role of fruits and vegetables in the diet of your child?
2.	Let's now discuss a different category of food: savory and sweet snacks. By "savory snacks", we usually refer to foods that are high in salt and are eaten before or after the main meals (e.g., crisps, savory crackers, cheese pie). Similarly, the term "sweet snacks" refers to foods that are high in sugar and are consumed before or after the main meals (e.g., chocolate, cakes, croissants, biscuits). Do you wish to talk to me about the role of savory and sweet snacks in the diet of your child?
3.	How about discussing the consumption of sugary drinks and juices? What do you think about the consumption of your child?
4.	Breakfast is the first meal of the day, eaten up to 2 hours after waking up. What is the role of breakfast in the diet of your child?
5.	Would you like to tell me about the mealtime habits of your family?
6.	For some households, the economic situation can affect the eating habits of the family, and in particular of the children. What is your opinion?
7.	Do you receive support and guidance from the government or other organizations to help your child adopt healthy eating habits (e.g., health promotion/information activities on healthy eating at school, in day care centers, financial assistance)? If so, by which organizations? What could help you in this regard?
8.	Some people think that school could help promote healthy eating among children. What is your opinion?
9.	Another important behavior, related to children's lifestyle, is the use of screens. Screen use is defined as the time spent using an electronic device, such as a mobile phone, computer, tablet, PlayStation, etc. Tell me about the role of these activities in the daily life of your child?
10.	Now let's talk about physical activity. By physical activity, we refer to activities during which the body moves, such as when playing sports or walking. What is the role of physical activity in the daily life of your child?

Table S2. Topic guides for conducting focus groups with Roma community representatives.

1.	Would you like to tell me about the role of fruits and vegetables in the diet of children in the community?
2.	Let's now discuss a different category of food, savory and sweet snacks. By "savory snacks" we usually refer to foods that are high in salt and are eaten before or after main meals (e.g., crisps, salty crackers, cheese pie). Similarly, the term "sweet snacks" refers to foods that are high in sugar and are consumed before or after main meals (e.g., chocolate, cakes, croissants, biscuits). Do you wish to talk to me about the role of salty and sweet snacks in the diet of children in the community?

3.	How about discussing the consumption of sugary drinks and juices? What do you think about the consumption of children in the community?
4.	Breakfast is the first meal of the day, eaten up to 2 hours after waking up. What place does breakfast have in the diet of children in the community?
5.	Would you like to tell me about children's mealtime habits?
6.	For some households, the economic situation can affect the eating habits of the family, especially children. What is your opinion?
7.	Do parents receive support and guidance from the government or other organizations to help children in the community adopt healthy eating habits (e.g., health promotion/information activities on healthy eating in the community, at school, financial assistance)? If so, by which organizations? What could help you in this regard?
8.	Some people think that school could help promote healthy eating for children. What is your opinion?
9.	Another important behavior, related to children's lifestyle, is the use of screens. Screen use is defined as the time spent with an electronic device, such as a mobile phone, computer, tablet, PlayStation, etc. Tell me about the role of these activities in the daily life of children in the community.
10.	Now let's talk about physical activity. By physical activity, we refer to activities during which the body moves, such as when playing sports or walking. What is the role of physical activity in the daily life of children in the community?

Table S3. Topic guides for conducting focus groups with caregivers in child protection units.

1.	Would you like to tell me about the role of fruits and vegetables in the diet of the children residing in the institution?
2.	Let's now discuss a different category of food, savory and sweet snacks. By "savory snacks" we usually refer to foods that are high in salt and are eaten before or after main meals (e.g., crisps, salty crackers, cheese pie). Similarly, the term "sweet snacks" refers to foods that are high in sugar and are consumed before or after main meals (e.g. chocolate, cakes, croissants, biscuits). Do you wish to talk to me about the role of savory and sweet snacks in the diet of the children residing in the institution?
3.	How about discussing the consumption of sugary drinks and juices? What do you think about the consumption of children residing in the institution?
4.	Breakfast is the first meal of the day, eaten up to 2 hours after waking up. What place does breakfast have in the diet of children residing in the institution?
5.	Would you like to tell me about children's mealtime habits?
6.	Some people think that the economic situation can affect the eating habits of the children residing in the institution. What is your opinion?
7.	Do you receive support and guidance from the government or other organizations to help children residing in the institution adopt healthy eating habits (e.g., health promotion/information activities on healthy eating in the institution, at school, financial assistance)? If so, by which organizations? What could help you in this regard?
8.	Some people think that school could help promote healthy eating for children. What is your opinion?
9.	Another important behavior, related to children's lifestyle, is the use of screens. Screen

	use is defined as the time spent with an electronic device, such as a mobile phone, computer, tablet, PlayStation, etc. Tell me about the role of these activities in the daily life of children.
10.	Now let's talk about physical activity. By physical activity, we refer to activities during which the body moves, such as when playing sports or walking. What is the role of physical activity in the daily life of children?

Table S4. Characteristics of children with disabilities. (N=45)

Age (years)	
Median (IQR)	10 (7, 15)
Gender N (%)	
Boys	29 (64%)
Girls	16 (36%)
Type of disability n (%)	
Autism Spectrum Disorder	25 (57%)
Cerebral palsy	11 (24%)
Intellectual Disabilities	4 (9%)
Loss of Vision	2 (4%)
Cri du chat syndrome	1 (2%)
Down's syndrome	1 (2%)
Dravet syndrome	1 (2%)
